# MicroRNA-363 targets myosin 1B to reduce cellular migration in head and neck cancer

**DOI:** 10.1186/s12885-015-1888-3

**Published:** 2015-11-06

**Authors:** Bhavana V. Chapman, Abigail I. Wald, Parvez Akhtar, Ana C. Munko, Jingjing Xu, Sandra P. Gibson, Jennifer R. Grandis, Robert L. Ferris, Saleem A. Khan

**Affiliations:** 1 Department of Microbiology and Molecular Genetics, University of Pittsburgh School of Medicine, Pittsburgh, PA 15219 USA; 2 Department of Immunology, University of Pittsburgh, Pittsburgh, PA 15216 USA; 3 Department of Otolaryngology, University of Pittsburgh and University of Pittsburgh Cancer Institute, Pittsburgh, PA 15213 USA; 4 Department of Pharmacology and Chemical Biology, University of Pittsburgh and University of Pittsburgh Cancer Institute, Pittsburgh, PA 15213 USA; 5 Present address: Clinical and Translational Science Institute,, Box 0558, 550 16th Street, 6th Floor, San Francisco, CA 94158 USA; 6 Medical Research Fellows Program, Howard Hughes Medical Institute, Chevy Chase, MD 20815 USA

**Keywords:** Squamous cell carcinoma of the head and neck, Human papillomavirus, miR-363, Myosin 1B

## Abstract

**Background:**

Squamous cell carcinoma of the head and neck (SCCHN) remains a prevalent and devastating disease. Recently, there has been an increase in SCCHN cases that are associated with high-risk human papillomavirus (HPV) infection. The clinical characteristics of HPV-positive and HPV-negative SCCHN are known to be different but their molecular features are only recently beginning to emerge. MicroRNAs (miRNAs, miRs) are small, non-coding RNAs that are likely to play significant roles in cancer initiation and progression where they may act as oncogenes or tumor suppressors. Previous studies in our laboratory showed that miR-363 is overexpressed in HPV-positive compared to HPV-negative SCCHN cell lines, and the HPV type 16-E6 oncoprotein upregulates miR-363 in SCCHN cell lines. However, the functional role of miR-363 in SCCHN in the context of HPV infection remains to be elucidated.

**Methods:**

We analyzed miR-363 levels in SCCHN tumors with known HPV-status from The Cancer Genome Atlas (TCGA) and an independent cohort from our institution. Cell migration studies were conducted following the overexpression of miR-363 in HPV-negative cell lines. Bioinformatic tools and a luciferase reporter assay were utilized to confirm that miR-363 targets the 3’-UTR of myosin 1B (MYO1B). MYO1B mRNA and protein expression levels were evaluated following miR-363 overexpression in HPV-negative SCCHN cell lines. Small interfering RNA (siRNA) knockdown of MYO1B was performed to assess the phenotypic implication of reduced MYO1B expression in SCCHN cell lines.

**Results:**

MiR-363 was found to be overexpressed in HPV-16-positive compared to the HPV-negative SCCHN tumors. Luciferase reporter assays performed in HPV-negative JHU028 cells confirmed that miR-363 targets one of its two potential binding sites in the 3’UTR of MYO1B. MYO1B mRNA and protein levels were reduced upon miR-363 overexpression in four HPV-negative SCCHN cell lines. Increased miR-363 expression or siRNA knockdown of MYO1B expression reduced Transwell migration of SCCHN cell lines, indicating that the miR-363-induced migration attenuation of SCCHN cells may act through MYO1B downregulation.

**Conclusions:**

These findings demonstrate that the overexpression of miR-363 reduces cellular migration in head and neck cancer and reveal the biological relationship between miR-363, myosin 1b, and HPV-positive SCCHN.

**Electronic supplementary material:**

The online version of this article (doi:10.1186/s12885-015-1888-3) contains supplementary material, which is available to authorized users.

## Background

Head and neck cancer ranks sixth amongst cancers worldwide and represents a heterogeneous collection of neoplasms, derived from the upper aerodigestive tract [1, 2]. The most common type, squamous cell carcinoma of the head and neck (SCCHN), occurs in the oral cavity, pharynx, and larynx. Tobacco use and alcohol consumption are the predominant risk factors for SCCHN. Recently, human papillomavirus (HPV) infection has been noted as an etiologic agent for a subset of SCCHN, specifically oropharyngeal malignancies, including tumors of the base of tongue and tonsil [3]. Despite advances in diagnosis and treatment, the five-year survival rate of 40–50 % has only incrementally improved in the last 20 years [4]. Prophylactic vaccination against high-risk HPV types may prevent a substantial number of oropharyngeal carcinomas in the future. However, the prolonged course of HPV carcinogenesis and the current prevalence of HPV-positive SCCHN warrant further investigation of its pathogenesis.

HPVs are small, circular, double-stranded, non-enveloped DNA viruses that infect the basal layer of squamous epithelial cells through abrasions or lesions in skin or mucosa. The relationship between high-risk HPV types (for example, HPV-16 and HPV-18) and cervical, anogenital, and oral cancers is well-established [5]. E6 and E7 oncoproteins from high-risk HPV types are essential for cellular transformation and functional inactivation of the tumor suppressor proteins p53 and retinoblastoma, respectively [1].

While the overall incidence of SCCHN has steadily declined, the prevalence of HPV-positive SCCHN cancers, specifically in developed nations, has markedly increased in the past decade due to a shift in sexual practices. SCCHN patient tumors from the United States demonstrate a 30 % HPV positivity rate [5], with more than 90 % of HPV-associated SCCHN due to HPV-16 [5]. HPV-positive and HPV-negative SCCHN demonstrate different clinical and demographic characteristics, leading some to classify them as distinct cancers [6]. HPV-positive SCCHN patients are generally younger men (40–55 years) from a higher socioeconomic status with minimal tobacco and alcohol exposure [5]. Tumors with a high viral load generally have a better prognosis compared to HPV-negative or low viral load tumors [7]. Despite late stage presentations due to early metastasis to secondary lymphoid tissues [8], HPV-positive tumors have a greater response to chemotherapy, radiation, and surgery [8]. HPV-positive tumors demonstrate improved immune system activation to viral antigens [9], lower recurrence rates [10], and more favorable disease outcomes [1] compared to HPV-negative head and neck tumors. While p53 contains inactivating mutations in more than half of HPV-negative oral cancers, it is rarely mutated in HPV-positive SCCHN [8]. The complex molecular mechanisms underlying the divergence in outcome depending on HPV-status is not well-understood. Elucidating the role of microRNAs (miRNAs, miRs) is one approach to evaluating the molecular differences between HPV-positive versus HPV-negative SCCHN.

MiRNAs are ~22 nucleotide-long, endogenously encoded, single-stranded RNAs that modulate the expression of over 50 % of human genes at the post-transcriptional level [11, 12]. MiRNAs are transcribed either from their own promoters or are processed from introns of protein-coding genes [13]. Precursor miRNAs (~70 nucleotides long) are exported into the cytoplasm where they are further processed into mature miRNAs and incorporated into the RNA-induced silencing complex (RISC) as a single strand [14]. RISC-associated miRNAs then pair with complementary sequences in the 3’-untranslated regions (UTRs) of one or more target mRNAs [14]. MiRNAs function as negative post-transcriptional regulators of gene expression by several mechanisms, including (1) site-specific cleavage of mRNAs; (2) enhanced mRNA degradation; and (3) inhibition of mRNA translation [14]. An mRNA may be targeted by several miRNAs while a single miRNA may target multiple mRNAs, thereby regulating dozens of genes.

MiRNAs regulate several cellular processes such as proliferation, differentiation, and apoptosis. Studies in chronic lymphocytic leukemia were the first to reveal the relationship between miRNAs and cancer pathogenesis in 2002 [15]. Since then, aberrant miRNA expression has been implicated in the initiation and progression of numerous hematological cancers and solid tumors, including SCCHN [16–18]. Notably, differential miRNAs expression profiles have been described in noncancerous versus tumor tissues [16, 19, 20]. MiRNAs may function as tumor suppressors or oncogenes depending on whether they target the mRNA of oncogenes or tumor suppressors, respectively. Consequently, confirming protein targets of a miRNA is critical in delineating their role in cancer. However, while several SCCHN miRNA profiling studies have been recently performed, the functional significance of dysregulated miRNAs in SCCHN is still poorly understood, especially in the context of HPV infection [20–31].

Although miR-363 is a member of the oncogenic miR-17 ~ 92 family of clusters [32], recent studies indicate that it may also possess tumor suppressor functions [33–36]. Thus, its role in oncogenesis is rather controversial at the present time. We previously reported miR-363 overexpression in HPV-positive SCCHN cell lines and showed that the HPV-16 E6 oncogene directly upregulates miR-363 [30]. In the current study, we have interrogated miR-363 expression in human SCCHN tumors and the phenotypic significance of miR-363 overexpression in SCCHN cell lines. MiRNA target prediction analysis suggested that miR-363 may target the 3’UTR of myosin 1B (MYO1B), a motor protein involved in cellular motility [37, 38]. Using luciferase reporter assays, we confirmed that miR-363 downregulates MYO1B expression in SCCHN cells by directly targeting the 3’UTR of MYO1B mRNA. By investigating the consequences of HPV-16 E6-mediated miR-363 expression in vitro, we aim to better resolve the clinicopathological features of HPV-positive SCCHN and identify prognostic markers of disease outcome, as well as new targets and therapeutic agents.

## Methods

### Patients

This study was approved by the Institutional Review Board of the University of Pittsburgh (protocol #99-069). Written informed consent was obtained from all patients. No children were enrolled in this study.

### Bioinformatics

MicroRNA data were extracted from The Cancer Genome Atlas (TCGA) Research Network (http://cancergenome.nih.gov/) portal isoform files for SCCHN tumors (accessed May 15, 2013). The reads per million miRNAs mapped data unit was evaluated, which represents each miRNA read count as a fraction of the total miRNA population for a particular tumor. Multiple reads from individual isoforms were combined into a single read count. The HPV-status of TCGA SCCHN tumors was noted according to the cBio Cancer Genomics Platform SCCHN database [39].

### SCCHN tumors

Forty-one SCCHN tissues were obtained from patients according to Institutional Review Board protocol #99–069. Upon surgical removal, a portion of all tissues was sent to pathology for tumor staging and the remainder was flash frozen until further processing.

### Cell lines and maintenance

The HPV-negative human SCCHN cell lines PCI13 and PCI30 were kindly provided by Dr. Theresa Whiteside (University of Pittsburgh Cancer Institute, Pittsburgh, PA) while the JHU028 and JHU029 cell lines were obtained from Dr. Joseph A. Califano (Johns Hopkins University School of Medicine, Baltimore, MD). PCI13 and PCI30 were cultured in Dulbecco's Modified Eagle's Medium (Lonza, Walkersville, MD) while JHU028 and JHU029 were cultured in RPMI-1640 (Lonza). All cells were supplemented with 10 % heat-inactivated FBS, 1 X penicillin/streptomycin solution (Lonza), and 2 mM L-glutamine (Gibco). Cell lines were maintained in a humidified cell incubator at 37 °C, 5 % CO_2_ atmosphere.

### Transfections

Cells were seeded to 50 % confluency in 6-well plates in antibiotic-free media 24 h prior to transfection. Cells were transfected with 50 nM premiR-363, negative premiR control (Applied Biosystems, Foster City, CA), or 50 nM small interfering RNA (siRNA) against MYO1B (ThermoFisher Scientific, San Jose, CA) using Lipofectamine 2000 (Invitrogen, Carlsbad, CA) and Opti-MEM^®^ (Gibco, Grand Island, NY). A FAM-labeled control premiR (Applied Biosystems) or a Block-it fluorescent oligonucleotide with no human homologous sequences (Invitrogen) was used as a control and to measure transfection efficiency in premiR and siRNA experiments, respectively. Cells were harvested 48 h after transfection and RNA and proteins were isolated for various assays as described below.

### DNA and RNA isolation

The Dneasy Blood & Tissue kit (Qiagen, Valencia, CA) was used to isolate DNA from SCCHN tissues according to the manufacturer’s protocol. Total RNA was isolated from SCCHN tissues and cell lines grown to 80-90 % confluency using the Ultraspec^TM^ RNA Isolation System (Biotecx, Houston, TX) according to the manufacturer’s protocol.

### HPV genotyping and quantitative real time RT-PCR

HPV status of the tumor tissues was assessed using the MY09/MY11 primer set, which amplifies a conserved region of the HPV L1 gene [40]. The glyceraldehyde-3-phosphate dehydrogenase (GAPDH) gene was used as a loading control using 5′-CGACCACTTTGTCAAGCTCA-3′ as the forward primer and 5′-AGGGGTCTACATGGCAACTG-3′ as the reverse primer. All PCR reactions were performed using 20 ng template DNA, 200 μM of each deoxynucleoside triphosphate (dNTP), 0.5 μM of each primer, and 0.5 units of Taq polymerase and the associated buffer (Promega, Madison, WI). Thermocycler conditions for all PCR reactions were 94 °C for 5 min; 35 cycles of 94 °C for 30 s, 57 °C for 30 s, and 72 °C for 30 s; and 72 °C for 10 min. The PCR-amplified DNA was visualized by agarose gel electrophoresis.

HPV-16 E6, HPV-16 E7, and MYO1B expression levels were measured by quantitative real-time RT-PCR (qRT-PCR) using the iTaq^TM^ Universal SYBR^®^ Green One-Step Kit (Bio-Rad) and the Real-Time thermocycler iQ5 (Bio-Rad, Hercules, CA, USA). The E6 forward primer 5′-AATGTTTCAGGACCCACAGG-3′, E6 reverse primer 5′-CAGCTGGGTTTCTCTACGTG-3′, E7 forward primer 5′-CATGGAGATACACCTACATTGCAT-3′, and E7 reverse primer 5′-GAACAGATGGGGCACACAAT-3′ were used to genotype HPV-positive SCCHN tissue samples. A 127 bp region of the MYO1B gene was amplified using the forward primer 5′-GGTCTGGTGTGGAGGTCCTA-3′ and the reverse primer 5′-CGTTGCTTCCTCAGGTCTTC-3′. HPV-16 E6, HPV-16 E7, and MYO1B mRNA levels were normalized to the GAPDH mRNA levels, using the forward primer 5′-CAGCCTCAAGATCATCAGCA-3′ and the reverse primer 5′-TGTGGTCATGAGTCCTTCCA-3′, amplifying a 106 bp region. DNase I-treated total RNA (60 ng) was used for each reaction, and all the reactions were performed in triplicate. Relative mRNA expression levels were calculated using the 2^-ΔΔCT^ values [41].

Mature miR-363 expression was confirmed by qRT-PCR using the TaqMan^®^ MicroRNA Reverse Transcription Kit and the TaqMan^®^ MicroRNA Assays (Applied Biosystems, Foster City, CA, USA) and the Real-Time thermocycler iQ5 (Bio-Rad, Hercules, CA, USA). The qRT-PCR experiments utilized stem-loop primers designed to amplify processed, mature miRNA. Total RNA (50 ng) was used for each reaction. All reactions were performed in triplicate according to the manufacturer’s instructions. MiRNA levels for each sample were normalized to small nucleolar (sno) RNU43 levels. Relative miRNA expression levels were calculated using the 2^-ΔΔCT^ values [41].

### Transwell migration assay

The HPV-negative SCCHN cell line, JHU028, was transfected with premiR-363 or an siRNA against MYO1B along with appropriate controls as described earlier. Forty-eight hours after transfection, cells were harvested and reseeded into 24-well 8 μM pore Transwell inserts (Corning) in serum-free media. The lower chambers of the Transwell plate were filled with 20 % FBS/RPMI media to serve as a chemoattractant. Transwells were stained with 0.1 % crystal violet at 1, 3, and 5 h following reseeding. Cells were photographed under the microscope and counted. The mean of eight fields from four separate trials was used to calculate the average number of migratory cells.

### Mutagenesis of the miR-363 binding sites in the MYO1B 3’ UTR

The MYO1B 3’UTR (1.4 kb; chr2:192,288,687-192,290,115) was PCR amplified using the forward primer 5’-GGACTAGTAACCGTCTCCTTGAAGTTGC-3’ and the reverse primer 5′-GGAAGCTTGGCACAAGGCAAGAAGAATC-3′. The primers were designed with a *SpeI* restriction site on the forward primer and a *HindIII* site on the reverse primer to aid in directional cloning of the amplified DNA into the pMIR-REPORT^TM^ vector (Applied Biosystems). The orientation of the inserted fragment was confirmed by restriction enzyme digestion and sequencing.

Deletion primers and the QuikChange XL Site-Directed Mutagenesis Kit (Agilent Technologies; Santa Clara, CA) was used to delete miR-363 binding site 1 (BS1) (chr2:192,288,731-192,288,738) or binding site 2 (BS2) (chr2: 192,289,618-192,289,625) from the 3’UTR of the MYO1B gene cloned into the pMIR-REPORT^TM^ vector (Applied Biosystems). BS1 was deleted using the forward primer 5’-CTACTTTCATGGACTTGTTCCTTTGTAATA-TGGTTTTGTTTTATTTGGGGTTCATTGTATG-3’ and the reverse primer 5’-CATACAATGAACCCCAAATAAAACAAAACCA-TATTACAAAGGAACAAGTCCATGAAAGTAG-3’. BS2 was deleted using the forward primer 5’-CCATTCAGATAGCAGTAAAACATTCTGTATGAT-AAACATCCAAGATCTTTTTTGAAAG-3’ and the reverse primer 5’-CTTTCAAAAAAGATCTTGGATGTTT-ATCATACAGAATGTTTTACTGCTATCTGAATGG-3’. Deletion mutants were confirmed by restriction enzyme digestion and DNA sequencing.

### Luciferase reporter assay

HPV-negative JHU028 cells were plated at 30,000 cells per well in 24-well plates (Corning). After 24 h, cells were transfected using Lipofectamine 2000 (Invitrogen) and Opti-MEM^®^ (Life Technologies). The pMIR-REPORT^TM^ MYO1B wild-type, 3’UTR BS1 or BS2 deletion constructs (500 ng) were co-transfected with 20 ng phRL-TK and 50 nM pre-miRs. All transfection experiments were repeated at least four times. Luciferase activity was measured 48 h post-transfection using the Dual Luciferase Reporter Assay System (Promega) according to manufacturer’s instructions and the Synergy 2 Luminometer (Biotek). RLU (Firefly/Renilla) activity was normalized to the MYO1B wild-type 3’ UTR co-transfected with phRL-TK only.

### Western blotting

Cells were lysed with radioimmunoprecipitation assay (RIPA) buffer at 4 °C directly on the 6 well-plate 48 h post-transfection with premiR-363 and the premiR negative control. Proteins (50 μg) from total cell lysates were separated on a 4-15 % SDS-polyacrylamide gradient gel (Bio-Rad) and transferred to Immobilon-P PVDF membrane (Millipore, Billerica, MA). After blocking, blots were incubated with a primary rabbit polyclonal antibody against MYO1B and a secondary anti-rabbit horseradish peroxidase antibody (both Santa Cruz Biotechnology, Santa Cruz, CA). A mouse monoclonal antibody against GAPDH (Chemicon, Billerica, MA) was used to normalize protein loading. Blots were visualized using the Western Lightning Plus ECL Substrate (Perkin Elmer; Waltham, MA), developed, and quantified by densitometry using AlphaView software by ProteinSimple (Santa Clara, CA).

### Statistical analysis

Statistical analysis was carried out using two-tailed t-tests. Data was considered significant at a value of *p* < 0.05.

## Results

### MiR-363 expression is significantly upregulated in head and neck tumors

**Fig. 1:**
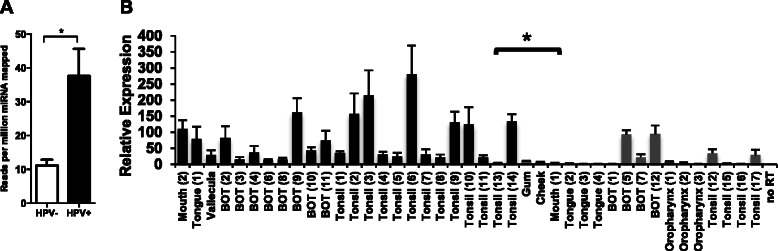
MiR-363 expression in SCCHN tissues **a** MiR-363 expression in The Cancer Genome Atlas SCCHN tumors. HPV-negative tumors possessed an average miR-363 reads per million miRNA mapped of 11.17 while HPV-positive tumors averaged a value of 37.58, indicating a 3.36-fold increase in miR-363 expression in HPV-positive tissues; *p < 0.001. **b** QRT-PCR analysis of miR-363 in HPV-positive and HPV-negative SCCHN tissues. Black bars, HPV-16-positive SCCHN samples; gray bars, HPV-negative SCCHN samples. Intensity values are relative to the HPV-negative SCCHN sample with the lowest miR-363 expression, which was arbitrarily assigned a value of 1; *p < 0.01 for HPV-positive samples compared to the HPV-negative samples; BOT, base of tongue; no RT, no reverse transcriptase added

Our analysis of 280 TCGA SCCHN tumors (245 HPV-negative and 35 HPV-positive) revealed that miR-363 expression is significantly increased (*p* < 0.001) in HPV-positive tumors compared to HPV-negative tumors (Figure 1a) [42]. We also examined an additional cohort of 41 SCCHN patients (24 HPV-positive and 17 HPV-negative tumors) treated at the University of Pittsburgh Cancer Institute (UPCI) between 2006 and 2009 (Fig. 1b, Table 1). Patient characteristics are further described in detail in Additional file 1. DNA PCR was performed to confirm the HPV status of tumor tissues (data not shown). HPV-positive SCCHN tissues expressed higher miR-363 levels compared to HPV-negative SCCHN samples as determined by qRT-PCR analysis (two-tailed *t*-test, *p* < 0.01; Fig. 1b). These data are consistent with our previous in vitro studies where HPV-positive SCCHN cell lines were found to have higher levels of miR-363 expression than the HPV-negative cell lines[30].

**Table 1:** Detailed demographics of SCCHN tissues

	HPV-16-positive	HPV-negative
Age, mean (SD)	55.6 (7.1)	64.2 (11.2)
Gender, *n* (%)		
Males	20 (83 %)	12 (71 %)
Females	4 (17 %)	5 (29 %)
Tumor location		
Mouth*	1	3
Tongue	1	3
Vallecula	1	0
Base of tongue	8	4
Oropharynx	0	3
Tonsil	13	4
Tumor stage		
T1NXMX	2	3
T2NXMX	21	5
T3NXMX	1	2
T4NXMX	0	6
Unknown	0	1

* Mouth samples include mouth, gum, and cheek

### Exogenous miR-363 suppresses the migratory ability of HPV-negative SCCHN cells

**Fig. 2:**
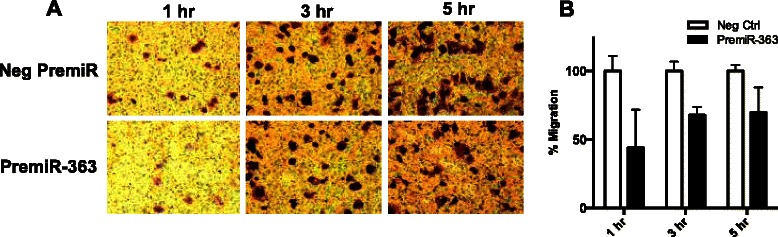
MiR-363 reduces the migratory ability of HPV-negative SCCHN cells **a** JHU028 cells transfected with premiR-363 exhibited decreased Transwell migration at 1, 3, and 5 h as compared to cells transfected with a negative premiR control. **b** Quantification of cell migration data from A. *p < 0.01

To determine the possible biological functions of miR-363, we transiently transfected HPV-negative SCCHN cell lines with premiR-363 or a negative premiR control and examined the effects on cell migration, proliferation, cell cycle and colony formation. Transwell migration assays showed a significant decrease in the number of migratory JHU028 cells overexpressing miR-363 compared to control cells at 1, 3, and 5 h (Fig. 2). Overexpression of miR-363 in HPV-negative SCCHN cell lines did not significantly affect cellular proliferation as examined by cell counting (Additional file 2), propidium iodide cell cycle (Additional file 3), and bromodeoxyuridine (BrdU; Additional file 4) cell cycle assays. Further, soft agar colony formation assays showed no difference in anchorage-independent growth between JHU028 cells overexpressing miR-363 and negative control cells (data not shown). Collectively, these results indicate that elevated miR-363 expression primarily reduces migration of SCCHN cell lines.

### MiR-363 targets the MYO1B 3’ UTR and reduces MYO1B expression

We have previously shown that 150 genes are downregulated in HPV-positive SCCHN tissues compared to HPV-negative SCCHN[43]. Since miRNAs negatively regulate their target genes, we identified potential miR-363 target genes using bioinformatic prediction tools (TargetScan, miRanda, Diana) [44–46]. A comparison of the putative target genes predicted by all three databases and the genes downregulated in HPV-positive cell lines identified 10 potential miR-363 target genes [43]. Of these, MYO1B was tested in our studies since its 3’ UTR contains two potential miR-363 binding sites (see Fig. 4a). Additionally, myosins are ubiquitous motor proteins involved in cellular processes such as motility [47–49].

**Fig. 3:**
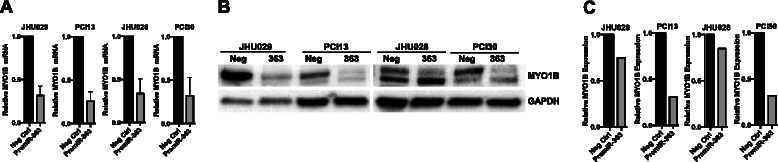
MiR-363 overexpression decreases MYO1B mRNA and protein expression **a** qPCR analysis for relative MYO1B mRNA levels following pre-miR-363 transfection as determined by the 2^-ΔΔCT^ method. Data were normalized to fold induction over negative control siRNA. **b** Western blot analysis of MYO1B and GAPDH protein expression. **c** Relative MYO1B:GAPDH levels obtained from densitometry of the blot shown in B

**Fig. 4:**
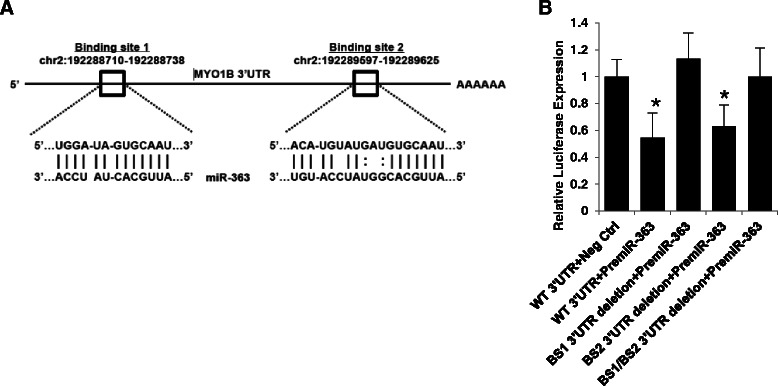
MiR-363 targets the 3’ UTR of MYO1B **a** Schematic representation of the 3’UTR of MYO1B with two complementary miR-363 target sites. Below, mature miR-363 sequences aligned to target sites as predicted by TargetScan, miRanda, and Diana. **b** MiR-363 targets binding site 1 within the MYO1B 3’-UTR. Four constructs were created that contained the wild-type MYO1B 3’ UTR, 3’ UTR with BS1 or BS2 deletion, and 3’ UTR with both BS1 and BS2 deletions, cloned downstream of the firefly luciferase gene in the pMiR-REPORT™ vector. Each of the constructs were transfected separately into JHU028 cells. MiR-363 significantly reduced firefly luciferase activity of pMiR-REPORT™ plasmid containing the wild-type MYO1B 3’ UTR or a plasmid lacking the BS2, implicating miR-363 binds to MYO1B 3’-UTR BS1 to suppress MYO1B expression; *p < 0.001 when compared to WT MYO1B 3’UTR

MYO1B expression at both the mRNA and protein levels was reduced upon miR-363 overexpression in four HPV-negative SCCHN cell lines (Fig. 3). A 68-73 % decrease in MYO1B mRNA expression was noted when miR-363 was overexpressed in SCCHN cell lines. However, there was no significant difference in MYO1B protein expression between HPV-positive vs. HPV-negative and primary vs. metastatic SCCHN tumors as detected by immunohistochemistry using an oropharyngeal tissue microarray (Additional file 5). To test whether MYO1B was a direct target of miR-363, luciferase reporter assays were performed in JHU028 cells with premiR-363 and reporter plasmids (with wild-type MYO1B 3’ UTR or the MYO1B 3’ UTR with BS1 or BS2 deletion, cloned downstream of the firefly luciferase gene in the pMiR-REPORT™ vector). As shown in Fig. 4b, miR-363 markedly reduced firefly luciferase activity of pMiR-REPORT™ plasmid containing the wild-type MYO1B 3’ UTR or a plasmid lacking the BS2. Taken together, these studies reveal that miR-363 binding site 1 (chr2:192,288,731-192,288,738) plays a functionally significant role in the regulation of MYO1B expression by this miRNA in head and neck cancer cell lines.

### siRNA knockdown of MYO1B reduces cell migration in HPV-negative SCCHN cells

**Fig. 5:**
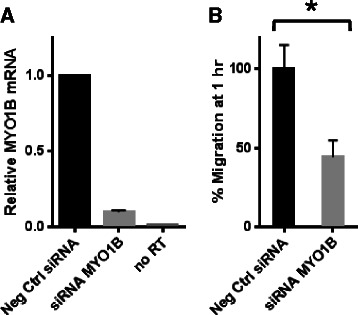
SiRNA knockdown of MYO1B reduces cell migration in HPV-negative SCCHN cells **a** qRT-PCR of MYO1B expression in JHU-028 48 h post-transfection. **b** Graph of migratory cells, normalized to negative Block-it control siRNA; *p < 0.01

In order to verify whether miR-363-mediated reduction in cellular migration is directly related to decreased MYO1B expression, JHU028 cells were transfected with an siRNA against MYO1B. As shown in Fig. 5a and b, knockdown of MYO1B via siRNA in HPV-negative SCCHN cells reduced Transwell migration compared to cells expressing a negative control siRNA. These effects were similar to those obtained with miR-363 overexpression, indicating that miR-363-induced migration attenuation of SCCHN cells acts through MYO1B downregulation.

## Discussion

SCCHN is a common and severe malignancy of the aerodigestive tract that is often diagnosed at late stages (III or IV). While advancements in surgery, chemotherapy, and radiation treatment have improved over time, patients often succumb to chemoradiation resistance, primary tumor invasion, metastasis, or recurrence. Notably, a number of studies indicate that HPV status is the strongest predictor of local recurrence, disease-specific survival, and overall survival in patients with SCCHN [50–52]. The heterogeneity of SCCHN malignancies contributes to the difficulty in treating patients with generic cancer protocols; personalized treatments based on tumor signatures may serve as an efficacious complement to current standards of care. Exploiting differences in miRNA expression between HPV-positive and HPV-negative SCCHN tissues and cell lines is one method of dissecting the complexity of tumor biology.

MicroRNAs are a rapidly developing field within cancer biology. MiRNA expression profiling studies have been reported in numerous solid tumor and hematological cancers. However, among noted deregulated miRNAs, few have been functionally validated and a limited number of potential targets have been confirmed. This gap in knowledge represents a significant roadblock in the development and application of miRNA-based cancer therapeutics.

Few HPV-specific SCCHN miRNA profiling studies have been performed to date. We previously reported 11 differentially expressed miRNAs between HPV-positive and HPV-negative SCCHN cell lines [30]. Of these, three (miR-363, −497, and −33) were up-regulated and eight were down-regulated (miR-155, 181a, 181b, −29 s, −218, −222, −221, and −142-5p) in HPV-positive cell lines. *Lajer* et al. reported similar findings with miR-363 as the most up-regulated miRNA in HPV-associated oropharyngeal cancer in a Danish population; miR-127-3p was the most down-regulated [27]. Comparisons between HPV-positive cervical and SCCHN tumor miRNA profiles reveal that the miR-15a/miR-16/miR195/miR-497 family, miR-143/miR-145 and the miR-106-363 cluster appear to be altered by HPV E6 and E7 oncoproteins [23].

MiR-363 is a member of the miR-17 ~ 92 family of clusters, which is composed of three clusters of miRNAs, miR-17-92 on chromosome 13, miR-106a-363 on chromosome X, and miR-106b-25 on chromosome 7, evolved through several deletions and duplications [53]. Since miRNAs from these clusters have similar or identical seed sequences, they potentially share protein targets. Thus, it has been hypothesized that miRNAs in the miR-17 ~ 92 family may possess related functions [32]. Researchers originally named the miR-17-92 polycistron “OncomiR-1” because the primary transcript represses *c-myc*-induced apoptosis in B-cell lymphomas [54]. Members of the OncomiR-1 family, miR-106a, −18b, −20b, −19b, −92-2, and −363 have been implicated in hematopoietic malignancies and solid tumors of the breast, colon, lung, pancreas, prostate, and stomach among others [16, 53, 55–59]. Conversely, the miR-17-92 cluster has also been found to exhibit tumor suppressive functions. Loss of heterozygosity at the 13q12-q13 region, where the miR-17-92 cluster is located, is linked with tumor progression and poor prognosis in a number of solid tumors, including SCC of the larynx, retinoblastoma, hepatocellular carcinoma, and nasopharyngeal carcinoma [60–65]. One study observed deletion of the miR-17-92 cluster in 16.5 % of ovarian cancers, 21.9 % of breast cancers, and 20.0 % of melanomas [66].

The precise role of miR-363 in tumorigenesis remains a controversial topic. MiR-363 has been noted to carry tumor suppressive properties in neuroblastoma, hepatocellular carcinoma, and colorectal cancer [34–36]. Recently, miR-363 has been reported to negatively regulate myeloid cell leukemia-1 (Mcl-2), an anti-apoptotic protein from the Bcl-2 family, and sensitize breast cancer cells to cisplatin [67]. Conversely, it has been shown to target pro-apoptotic caspases in glioblastoma, thereby acting as an oncomiR [68]. We speculate that the role of miRNAs as oncogenes or tumor suppressors may be tissue-specific. The targets of a particular miRNA may vary in different cell types and tissues based on the expression levels of the miRNA and its potential mRNA targets, differential expression of mRNA binding proteins, and alternative processing of mRNAs [69, 70]. Thus, it is not always feasible to extrapolate a miRNA’s functional role based on its cluster or its implication in a different tissue type.

After reporting miR-363 upregulation in HPV-positive cell lines in our previous study [30], we sought to corroborate our findings in SCCHN tumors In the present study, we confirmed miR-363 overexpression in HPV-positive tumors in TCGA and the UPCI patient cohorts. Our functional phenotypic studies revealed that miR-363 inhibits SCCHN cell migration and invasion, in part, due to inhibition of MYO1B expression. We also performed the well-established luciferase reporter assay, to confirm MYO1B as miR-363 target. A correlative decrease in mRNA and protein levels of MYO1B was observed following miR-363 expression. Similarly, *Sun* et al. correlated decreased miR-363 levels in SCCHN tumor tissues with increased rates of sentinel lymph node metastasis, though HPV status was not examined [33]. This group identified another miR-363 target, podoplanin (PDPN), a protein involved in cell motility [33]. Taken together, these results suggest that the overexpression of miR-363, and subsequent decrease in MYO1B and PDPN, is one pathway by which metastasis may be reduced in HPV-positive SCCHN.

Despite having a milder clinical course, HPV-positive, p16-positive SCCHN tends to have increased nodal metastasis, but comparable rates of distant metastasis versus HPV-negative SCCHN [71, 72]. The mechanism underlying this phenomenon is yet to be elucidated. Some studies note that HPV-positivity is a factor associated with poorly differentiated tumors, which may, result in early lymphatic spread [73–75]. The heterogeneity of head and neck tumors in combination with HPV infection may cause complex molecular perturbations in the cell and differences in survival and migration pathways that are affected may affect chemotherapy or radiation resistance and therefore prognosis of HPV-positive vs. HPV-negative SCCHN. Finally, we also delineate a link between miR-363 and MYO1B in the setting of HPV-positive SCCHN.

## Conclusion

In summary, we have shown that miR-363 is upregulated in HPV-positive SCCHN tumors. Through in vitro models, we have demonstrated that miR-363 decreases migration of SCCHN cells by targeting myosin 1B, a motility protein. The clinical relevance of increased miR-363 and diminished MYO1B expression in HPV-positive SCCHN will be the subject of future investigation.

## Additional files

Additional file 1: **Detailed demographics of SCCHN tissues.** (DOCX 135 kb)
Additional file 2: **Cell counting assay.** (PPTX 162 kb)
Additional file 3: **Cell cycle analysis using propidium iodide DNA staining.** (PPTX 54 kb)
Additional file 4: **Cell cycle analysis using bromodeoxyuridine (BrdU) staining.** (PPTX 59 kb)
Additional file 5: **MYO1B immunohistochemistry of SCCHN primary and metastatic tumor tissue microarray.** (PPTX 113 kb)

## Abbreviations

BOT: base of tongue
HPV: human papillomavirus
miRNA, miR: microRNA
MYO1B: myosin 1B
No RT: no reverse transcriptase added
PCR: polymerase chain reaction
RISC: RNA-induced silencing complex
SCCHN: squamous cell carcinoma of the head and neck
siRNA: small interfering RNA
TCGA: The Cancer Genome Atlas
