# Supplementary material for: MicroRNA-363 targets myosin 1B to reduce cellular migration in head and neck cancer
Source: BMC Cancer. 2015 Nov 6;15:861. doi: 10.1186/s12885-015-1888-3 (PMC4635687; doi:10.1186/s12885-015-1888-3)
Supplement: Additional file 1: — Detailed demographics of SCCHN tissues. (DOCX 135 kb) [file 12885_2015_1888_MOESM1_ESM.docx]

**Additional File 1: Detailed demographics of SCCHN tissues**

| **Specimen Site** | **TNM Stage** | **Race** | **HPV status** | **Tobacco history** | **Alcohol history** | **Status** | **Metastasis** |
| --- | --- | --- | --- | --- | --- | --- | --- |
| Gum | T1N0M0 | Caucasian | HPV-negative | never smoker | never drinker | Deceased | cheek mucosa, mouth, tongue |
| Cheek | T2N1M0 | Caucasian | HPV-negative | former smoker | current drinker | Deceased | cheek mucosa, lip, soft palate, oropharynx |
| Mouth (1) | T1N1M0 | Caucasian | HPV-negative | never smoker | never drinker | Alive | none |
| Mouth (2) | T2N2CM0 | Caucasian | HPV-16 | former smoker | former drinker | Alive | none |
| Tongue (1) | T3N2M0 | Caucasian | HPV-16 | current smoker | current drinker | Deceased | mouth, lung |
| Tongue (2) | T3N2BM0 | Caucasian | HPV-negative | current smoker | former drinker | Deceased | none |
| Tongue (3) | T4N2BM0 | Caucasian | HPV-negative | current smoker * | current drinker * | Deceased | none |
| Tongue (4) | T4AN2CM0 | Caucasian | HPV-negative | former smoker | current drinker | Deceased | none |
| Vallecula | T2BN2M0 | African-American | HPV-16 | never smoker | never drinker | Alive | none |
| Base of tongue (1) | T1N2BM0 | Caucasian | HPV-negative | current smoker * | current drinker | Deceased | none |
| Base of tongue (2) | T1N2BM0 | Caucasian | HPV-16 | current smoker * | former drinker | Alive | none |
| Base of tongue (3) | T2N0 | Caucasian | HPV-16 | former smoker | never drinker | Deceased | lymph node (tongue) |
| Base of tongue (4) | T2N0M0 | Caucasian | HPV-16 | former smoker | never drinker | Deceased | none |
| Base of tongue (5) | T2N0M0 | Caucasian | HPV-negative | former smoker | current drinker * | Deceased | base of tongue |
| Base of tongue (6) | T2N1M0 | Caucasian | HPV-16 | current smoker * | former drinker | Alive | none |
| Base of tongue (7) | T2N1M0 | Caucasian | HPV-negative | current smoker | current drinker * | Alive | lung |
| Base of tongue (8) | T2N2M0 | Caucasian | HPV-16 | unknown | unknown | Deceased | recurrence - vallecula |
| Base of tongue (9) | T2N2BM0 | Caucasian | HPV-16 | never smoker | never drinker | Alive | none |
| Base of tongue (10) | T2N2CM0 | Caucasian | HPV-16 | current smoker | current drinker | Deceased | lymph nodes (head/face/neck) |
| Base of tongue (11) | T2N3M0 | African-American | HPV-16 | current smoker * | current drinker | Deceased | lymph nodes (intrathoracic) |
| Base of tongue (12) | T3N0M0 | Caucasian | HPV-negative | never smoker | never drinker | Alive | none |
| Oropharynx (1) | T2N0M0 | Caucasian | HPV-negative | current smoker | former drinker | Alive | none |
| Oropharynx (2) | T4BN2BM0 | Caucasian | HPV-negative | former smoker | current drinker | Deceased | tongue, lymph nodes (head/face/neck) mouth |
| Oropharynx (3) | recurrent - stage unknown | Caucasian | HPV-negative | current smoker * | current drinker * | Alive | primary - gum, 2 recurrences oropharynx |
| Tonsil (1) | T1N1M0 | Caucasian | HPV-16 | never smoker | current drinker | Alive | met via PET scan - Level II LN |
| Tonsil (2) | T2N0M0 | Caucasian | HPV-16 | unknown | unknown | Alive | none |
| Tonsil (3) | T2N0M0 | Caucasian | HPV-16 | former smoker | never drinker | Alive | recurrence - base of tongue |
| Tonsil (4) | T2N0M0 | Caucasian | HPV-16 | never smoker | never drinker | Alive | none |
| Tonsil (5) | T2N1M0 | Caucasian | HPV-16 | snuff * | current drinker | Deceased | bones in skull/face, lung, bones in pelvic/sacrum |
| Tonsil (6) | T2N2 | Caucasian | HPV-16 | former smoker | never drinker | Alive | none |
| Tonsil (7) | T2N2M0 | Caucasian | HPV-16 | current smoker | current drinker | Deceased | none |
| Tonsil (8) | T2N2AM0 | Caucasian | HPV-16 | former smoker | former drinker | Alive | none |
| Tonsil (9) | T2N2BM0 | Caucasian | HPV-16 | current smoker | current drinker | Alive | none |
| Tonsil (10) | T2N2BM0 | Caucasian | HPV-16 | current smoker * | current drinker | Alive | none |
| Tonsil (11) | TXN2BM0 | Caucasian | HPV-16 | former smoker | current drinker | Alive | none |
| Tonsil (12) | T2N2BM0 | Caucasian | HPV-negative | current smoker * | current drinker * | Alive | none |
| Tonsil (13) | T2N2CM0 | Caucasian | HPV-16 | current smoker * | current drinker * | Alive | none |
| Tonsil (14) | T2N3M0 | Caucasian | HPV-16 | current smoker * | current drinker * | Alive | none |
| Tonsil (15) | T4N2BM0 | Caucasian | HPV-negative | current smoker * | current drinker * | Deceased | none |
| Tonsil (16) | T4N3M0 | Caucasian | HPV-negative | current smoker * | former drinker | Deceased | none |
| Tonsil (17) | T4AN2BM0 | Caucasian | HPV-negative | current smoker | current drinker | Deceased | none |

Numbers in parenthesis correspond to the sample numbers described in Figures 1B of the article.

Abbreviation: *Unknown if quit tobacco and alcohol use.
