# Supplementary material for: MicroRNA-363 targets myosin 1B to reduce cellular migration in head and neck cancer
Source: BMC Cancer. 2015 Nov 6;15:861. doi: 10.1186/s12885-015-1888-3 (PMC4635687; doi:10.1186/s12885-015-1888-3)
Supplement: Additional file 2: — Cell counting assay. (PPTX 162 kb) [file 12885_2015_1888_MOESM2_ESM.pptx]

## Slide 1
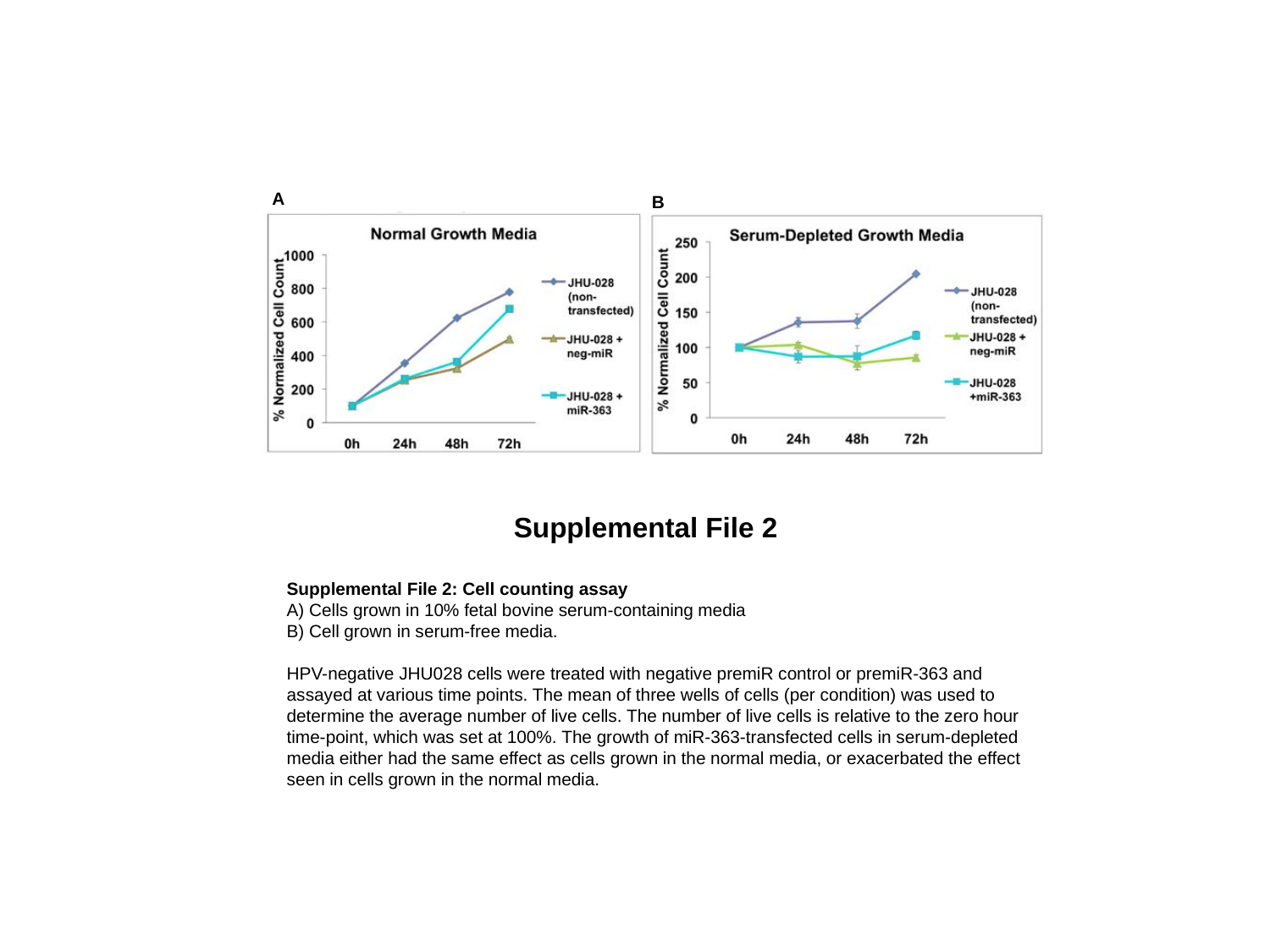

A
B
Supplemental File 2
Supplemental File 2: Cell counting assay
A) Cells grown in 10% fetal bovine serum-containing media
B) Cell grown in serum-free media.
HPV-negative JHU028 cells were treated with negative premiR control or premiR-363 and assayed at various time points. The mean of three wells of cells (per condition) was used to determine the average number of live cells. The number of live cells is relative to the zero hour time-point, which was set at 100%. The growth of miR-363-transfected cells in serum-depleted media either had the same effect as cells grown in the normal media, or exacerbated the effect seen in cells grown in the normal media.
