# Supplementary material for: MicroRNA-363 targets myosin 1B to reduce cellular migration in head and neck cancer
Source: BMC Cancer. 2015 Nov 6;15:861. doi: 10.1186/s12885-015-1888-3 (PMC4635687; doi:10.1186/s12885-015-1888-3)
Supplement: Additional file 3: — Cell cycle analysis using propidium iodide DNA staining. (PPTX 54 kb) [file 12885_2015_1888_MOESM3_ESM.pptx]

## Slide 1
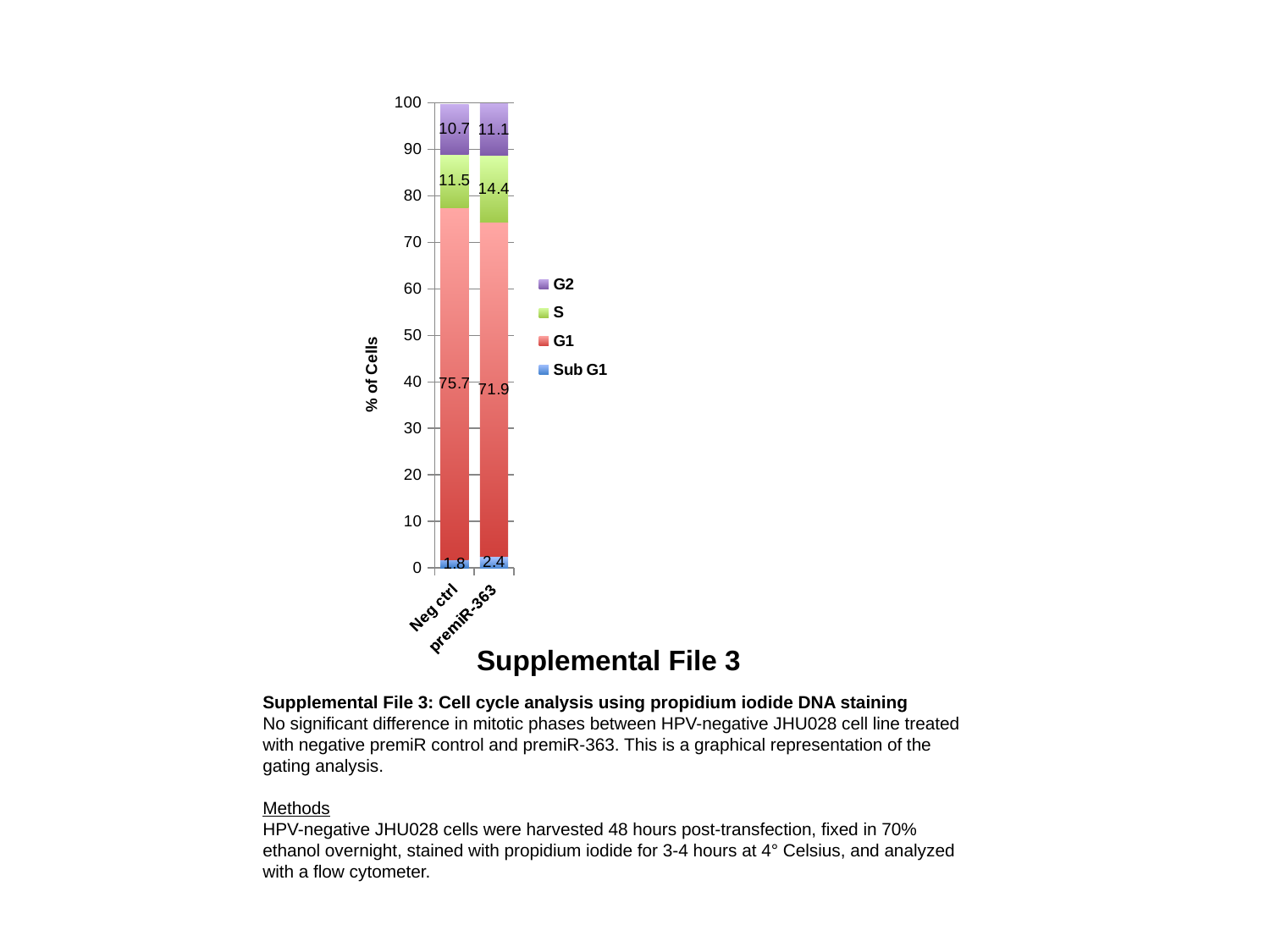

### Chart
| Category | Sub G1 | G1 | S | G2 |
|---|---|---|---|---|
| Neg ctrl | 1.8 | 75.7 | 11.5 | 10.7 |
| premiR-363 | 2.4 | 71.9 | 14.4 | 11.1 |Supplemental File 3
Supplemental File 3: Cell cycle analysis using propidium iodide DNA staining
No significant difference in mitotic phases between HPV-negative JHU028 cell line treated with negative premiR control and premiR-363. This is a graphical representation of the gating analysis.
Methods
HPV-negative JHU028 cells were harvested 48 hours post-transfection, fixed in 70% ethanol overnight, stained with propidium iodide for 3-4 hours at 4° Celsius, and analyzed with a flow cytometer.
