# Supplementary material for: MicroRNA-363 targets myosin 1B to reduce cellular migration in head and neck cancer
Source: BMC Cancer. 2015 Nov 6;15:861. doi: 10.1186/s12885-015-1888-3 (PMC4635687; doi:10.1186/s12885-015-1888-3)
Supplement: Additional file 4: — Cell cycle analysis using bromodeoxyuridine (BrdU) staining. (PPTX 59 kb) [file 12885_2015_1888_MOESM4_ESM.pptx]

## Slide 1
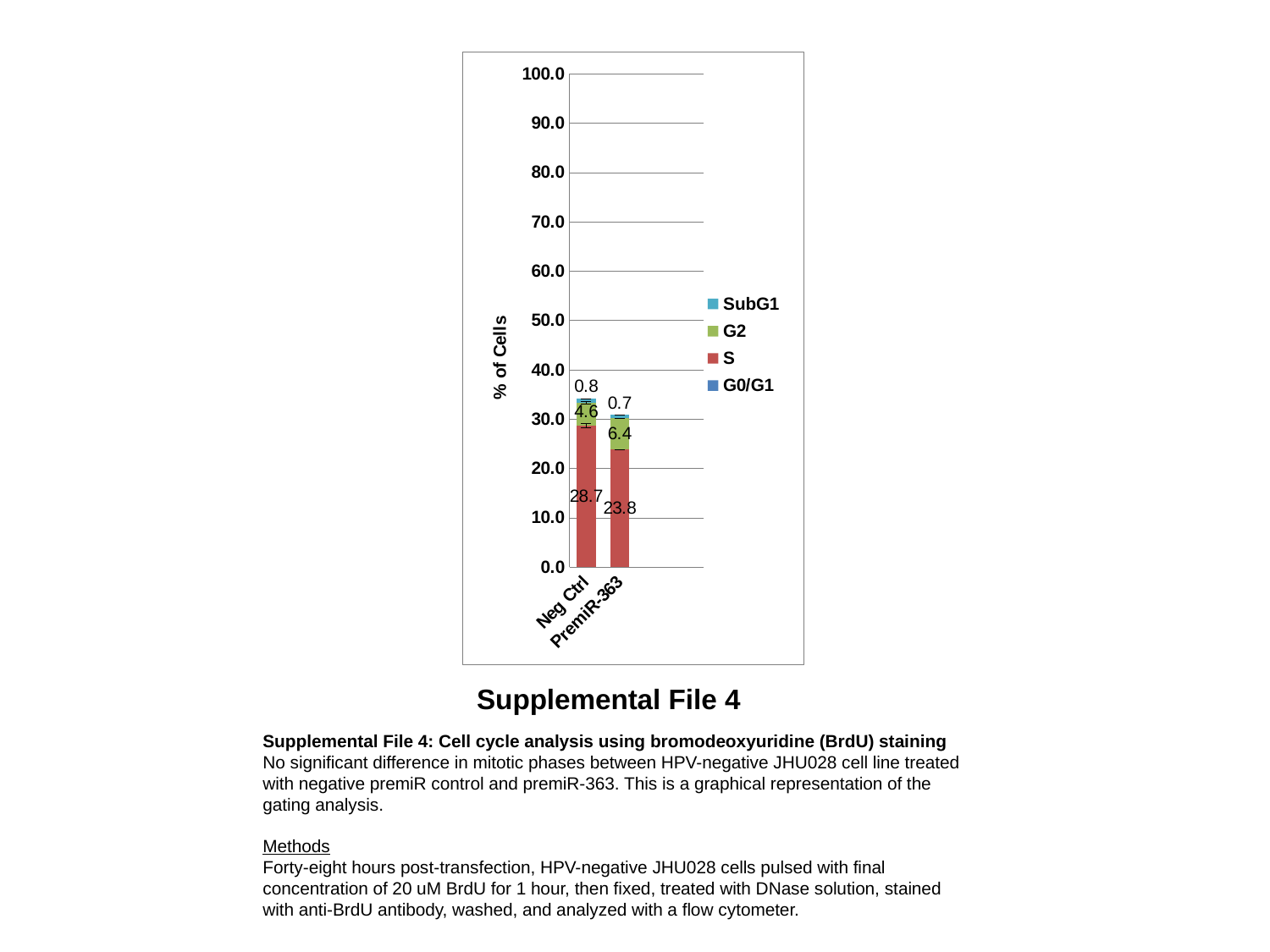

### Chart
| Category | G0/G1 | S | G2 | SubG1 |
|---|---|---|---|---|
| Neg Ctrl | 64.7333333333333 | 28.73333333333328 | 4.609999999999998 | 0.754 |
| PremiR-363 | 67.93333333333334 | 23.8 | 6.39 | 0.706 |Supplemental File 4
Supplemental File 4: Cell cycle analysis using bromodeoxyuridine (BrdU) staining
No significant difference in mitotic phases between HPV-negative JHU028 cell line treated with negative premiR control and premiR-363. This is a graphical representation of the gating analysis.
Methods
Forty-eight hours post-transfection, HPV-negative JHU028 cells pulsed with final concentration of 20 uM BrdU for 1 hour, then fixed, treated with DNase solution, stained with anti-BrdU antibody, washed, and analyzed with a flow cytometer.
