# Supplementary material for: MicroRNA-363 targets myosin 1B to reduce cellular migration in head and neck cancer
Source: BMC Cancer. 2015 Nov 6;15:861. doi: 10.1186/s12885-015-1888-3 (PMC4635687; doi:10.1186/s12885-015-1888-3)
Supplement: Additional file 5: — MYO1B immunohistochemistry of SCCHN primary and metastatic tumor tissue microarray. (PPTX 113 kb) [file 12885_2015_1888_MOESM5_ESM.pptx]

## Slide 1
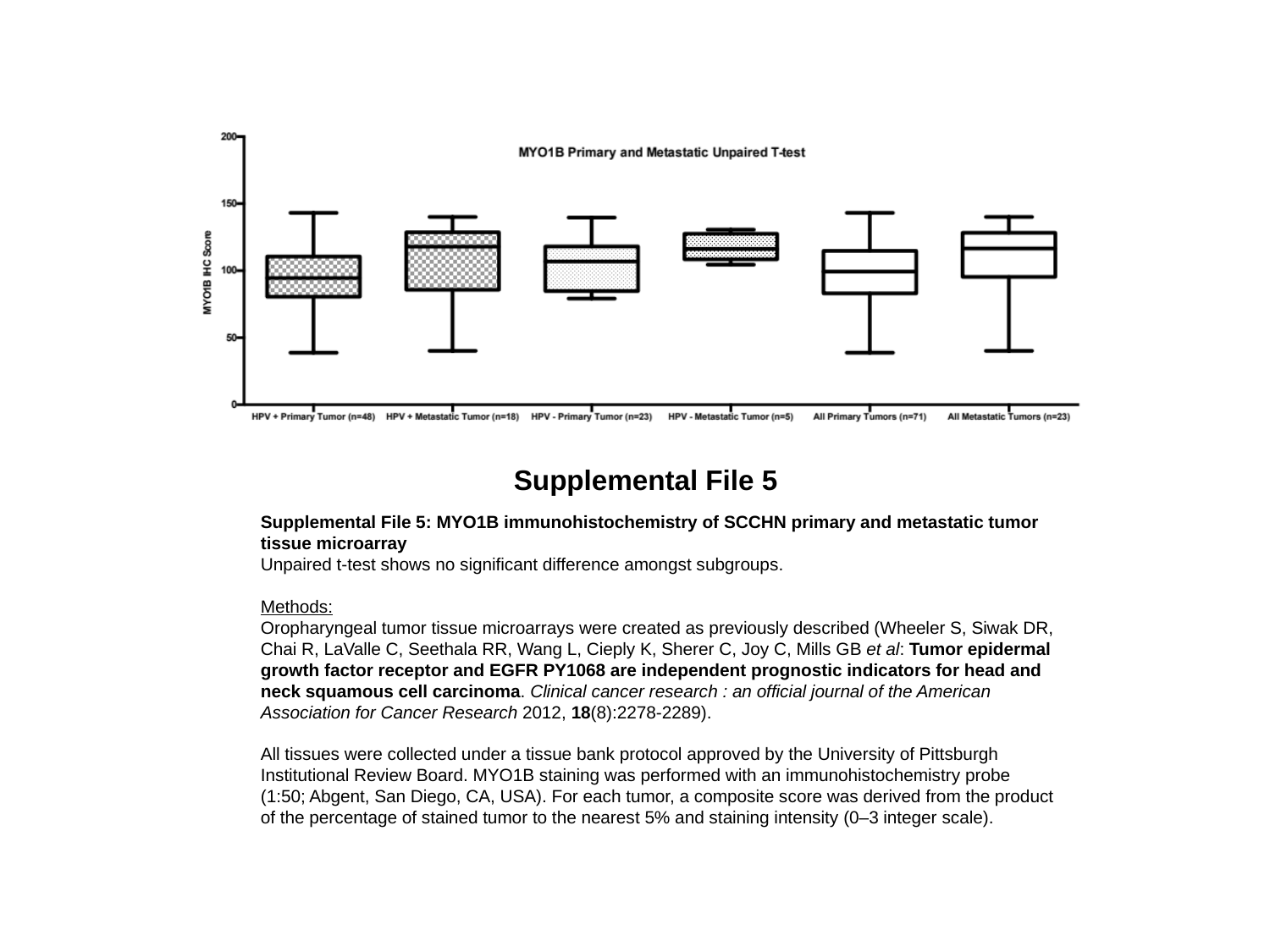

Supplemental File 5
Supplemental File 5: MYO1B immunohistochemistry of SCCHN primary and metastatic tumor tissue microarray
Unpaired t-test shows no significant difference amongst subgroups.
Methods:
Oropharyngeal tumor tissue microarrays were created as previously described (Wheeler S, Siwak DR, Chai R, LaValle C, Seethala RR, Wang L, Cieply K, Sherer C, Joy C, Mills GB et al: Tumor epidermal growth factor receptor and EGFR PY1068 are independent prognostic indicators for head and neck squamous cell carcinoma. Clinical cancer research : an official journal of the American Association for Cancer Research 2012, 18(8):2278-2289).
All tissues were collected under a tissue bank protocol approved by the University of Pittsburgh Institutional Review Board. MYO1B staining was performed with an immunohistochemistry probe (1:50; Abgent, San Diego, CA, USA). For each tumor, a composite score was derived from the product of the percentage of stained tumor to the nearest 5% and staining intensity (0–3 integer scale).
